# Physical Activity, Public Policy, Health Promotion, Sociability and Leisure: A Study on Gymnastics Groups in a Brazilian City Hall

**DOI:** 10.3390/ijerph20085516

**Published:** 2023-04-14

**Authors:** Caroline Giolo-Melo, Reinaldo Tadeu Boscolo Pacheco

**Affiliations:** School of Arts, Sciences and Humanities, University of São Paulo—USP, Rua Arlindo Béttio, 1000-Ermelino Matarazzo, Sao Paulo 03828-000, Brazil

**Keywords:** physical activity, gymnastics, fitness, public policy, quality of life, health promotion, leisure, sociability, focus group

## Abstract

Background: The present study analyzed a gymnastics program offered by the Department of Sports and Leisure at a Brazilian city hall, representing an example of an effective and consolidated public policy. Main objective: The objective of this study was to understand the reasons for enrollment/joining (adhesion) and permanence (adherence) among female students in gymnastics groups in order to understand why this public policy has been taking place uninterruptedly for over thirty years and to characterize the quality of life of female gymnastics students. Methods: This is a case study that used mixed methods. As a quantitative method, the Portuguese version of the World Health Organization Quality of Life—Abbreviated Version (WHOQOL-bref) questionnaire was used. As a qualitative method, the focus group technique was used. Thus, 239 women aged between thirty-five and seventy-four years old, who were students of the gymnastics program, participated in this research by answering the WHOQOL-bref questionnaire. Two classes were selected using the socioeconomic criterion of social classes in inverse situations to carry out one focus group session; then, twenty students belonging to the two classes were randomly selected. Results: It was verified that the public policy studied has led to an improvement in the quality of life of the students, with the benefits extending beyond physical health, and that in both groups adhesion occurred mainly due to the indication of students who already practiced, or due to medical indications. It was also found that, in both groups, the main reason for long-term adherence was the understanding of the gymnastics class as a space for sociability and a moment of leisure. Conclusion: Physical activity is an important health promotion strategy. In addition to its biological benefits, which are necessary for the prevention of chronic non-communicable diseases, it also improves health and quality of life through social and psychological benefits, characterizing it as an effective health care strategy from a biopsychosocial perspective.

## 1. Introduction

Health promotion is a trend that emerged in the early 1980s, which has come to represent a unifying concept for those who recognize the need to change their ways and conditions of life in order to promote health. In this way, it represents a mediating strategy between people and their environments, synthesizing personal choice and social responsibility in health to create a healthier future. The basic resources for health are income, shelter and food [1]. The Ottawa Charter is considered the founding document of health promotion and was presented at the end of the First International Conference on Health Promotion, held in Ottawa, Canada, in 1986. It is a letter of intent that seeks to contribute to health policies in all countries, equally and universally [2]. Thus, it refers to a social right, inherent to the condition of citizenship, requiring public policies to guarantee the population’s access to it.

However, improving health requires not only a secure foundation in these basic resources but also information and life skills, supportive environments (providing opportunities to make healthy choices concerning properties, services and facilities) and conditions in economic environments, and physical, social and cultural factors (the “total” environment) that positively influence health [1]. The World Health Organization (WHO) defines health as a state of complete physical, mental and social well-being and not merely the absence of disease or infirmity. Thus, health promotion focuses on all the conditions required, not only to prevent disease and prolong life expectancy, but also to improve the quality of life (QoL), resulting from a set of social, economic, political and cultural factors.

From this perspective, municipal public policies for sport and leisure emerge, as they are configured as catalysts for a healthy lifestyle, where they guarantee the population’s access to regular physical activity, providing people with a physical and mental balance, which is very important for promoting health and quality of life. However, for a public policy to be effective, it is essential that there is enrollment (adhesion) and permanence (adherence) in the practices; thus, it is necessary to consider the needs of the public served during policy formulation, and to understand the reasons for the adherence and permanence of students, so that policies can be improved.

Additionally, health promotion, understood as a strategy for the social production of health, must articulate and permeate public policies that influence the future of urban quality of life [3], such as the public policy presented in this study. The studied public policy consists of gymnastics classes performed in groups offered by the Department of Sports and Leisure (Secretaria de Esportes e Lazer, SEL) of the Municipality of Valinhos, State of São Paulo, Brazil, which have been taking place uninterruptedly since the mid-1980s, that is, they have been happening for about thirty years, despite all the changes in municipal management that have taken place. This policy was chosen for the present study due to its characteristic of being an effective public policy, representing an important example for the area.

At this point, it is essential to emphasize that guaranteeing the provision of sports and leisure to citizens is a duty of the public authorities and must be provided through social public policies and concrete actions that can ensure the minimum conditions for access to and permanence in sports programs and leisure activities. In this way, not only are public spaces democratized, but society is also encouraged to get involved in sport and leisure practices [4]. Bearing in mind that minimally effective state interventions help to perpetuate, maintain or expand inequalities in access [5], public sport and leisure policies must also be democratic and inclusive, in addition to not being limited to isolated events or directed solely to the constitution of sports competition calendars, which serve only a portion of society.

In this perspective, it should be mentioned that the present article refers to a master’s thesis entitled “Public policy and quality of life: a study about the gymnastics groups of the City Hall of Valinhos—SP” [6], carried out in the Department of Sports and Leisure of the City Hall of the Municipality of Valinhos, State of São Paulo, Brazil, which was presented and defended at the School of Arts, Sciences and Humanities of the University of São Paulo (USP) in 2019. The main objective of the research was to understand the reasons for the students’ enrollment/joining (adhesion) and permanence (adherence) to gymnastics groups, in order to understand why this public policy has been taking place uninterruptedly for over thirty years, and to characterize the students’ quality of life. The secondary objectives were to identify what this participation means for them, to understand the strengths and weaknesses of the gymnastics program, and to identify what quality of life means for the students and whether they use other sport and leisure facilities or carry out other guided or autonomous physical activities. As a hypothesis, we believe that adhesion to the gymnastics program occurs mainly due to medical indications and that the practice of gymnastics provides an improvement in physical abilities and biological indicators, directly reflecting the quality of life of the students, as well as a moment of leisure, which leads to long-term adherence.

## 2. Materials and Methods

### 2.1. Study Design

This is a case study that used mixed methods—qualitative and quantitative—in order to obtain broader perspectives to achieve the proposed objectives and a closer view of the reality of the gymnastics modality as a municipal public policy. The Portuguese version of the World Health Organization Quality of Life—Abbreviated Version (WHOQOL-bref) [7] questionnaire was used for all participants, and the focus group technique was used in two classes which were selected using the criterion of social classes in inverse situations.

### 2.2. Research Subjects

**Figure 1:**
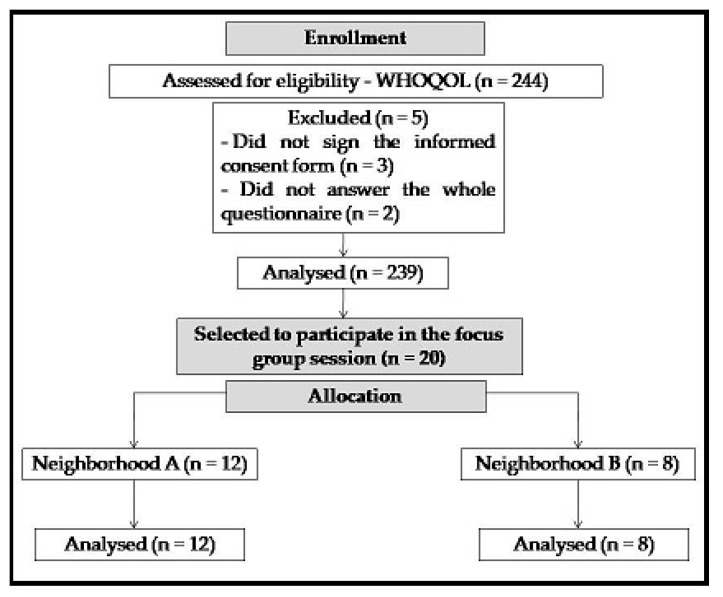
Study flowchart.

The participants were 239 women aged between thirty-five and seventy-four years old, who were students of the SEL’s gymnastics classes and were randomly selected according to availability. Two classes were selected using the socioeconomic criterion of social classes in inverse situations to carry out one focus group session; then, twenty students belonging to the two classes were randomly selected. Inclusion criteria were as follows: students of an SEL gymnastics group, who were willing to participate in this study, completed the WHOQOL-bref questionnaire, signed the informed consent form, participated in the focus group, belonged to one of the two classes selected and attended the program assiduously (75% attendance). Exclusion criteria were as follows: students who did not sign the free informed consent form or who did not answer the entire questionnaire. The study flowchart is shown in Figure 1.

### 2.3. Quantitative Instrument

To assess quality of life, the Portuguese version of the World Health Organization Quality of Life—Abbreviated Version (WHOQOL-bref) [7] questionnaire was used, which consists of 26 questions, that is, 2 general questions on quality of life and 24 on the following domains: physical, psychological, social relations and environment. According to the sample size calculation, the minimum size required was 204 participants. For the calculation, the total population of 800 students who currently participate in the gymnastics program was used, considering a margin of error of 5% and a confidence level of 90%. An increase of 20% in the minimum size was calculated, so 244 questionnaires were answered; however, 3 questionnaires were excluded before tabulation, as the students did not sign the free informed consent form, and 2 were tabulated but invalidated by the tool because the students failed to answer a whole page of the questionnaire. Thus, according to the size of the final sample of valid responses, 239 current students of the gymnastics program, aged between thirty-five and seventy-four years old, participated in the research. The data collection process was completed in 5 weeks, and the students were invited to participate in the research at the time of the gymnastics class.

### 2.4. Qualitative Instrument

The focus group technique is a qualitative method that employs interviews on a specific topic among a small group of people, usually homogeneous, called a focus group. In this technique, the researcher can gather information about several individuals in a single session [8]. Regarding the number of participants in the focus groups, the literature recommends between 6 and 15 participants [9,10]. Table 1 presents the background information of the focus group participants.

In this study, one focus group session was carried out in two classes (Neighborhood A and Neighborhood B (n = 20) which were selected using the criterion of social classes in inverse situations. This criterion is a socioeconomic criterion that refers to two distinct groups with clear class divisions. This criterion was chosen since one group lived in a more privileged region; therefore, this division represented—if not an “inverse” situation—at least a “diverse” situation in terms of income, housing, mobility, education, access to health care, etc. We chose to carry out the focus group in only two gym classes, instead of all of the classes, since all of the classes were similar and conducted in the same way, as was the case in a previous study [11] (which used the discourse analysis technique in the answers to a semi-structured questionnaire about what it means for the students to participate in gymnastics classes). It was verified that the answers were similar in the different groups, meaning it would have been unnecessary to carry out the collection in all of the classes; therefore, this form of selection was chosen to also understand whether economic and social differences influenced the responses. Twenty students, aged between forty-four and sixty-five, who were randomly selected according to availability, participated in this stage.

A pre-established script elaborated by the authors was used as a guiding scheme, with the main information to facilitate the progress of data collection, as well as practical guidelines for carrying out the focus groups. The questions used were divided into three sections: personal history of the students; gymnastics program; and general questions. The questions on the students’ personal history related to the following: age and time of participation in the gymnastics group; practice of physical activity in childhood and adolescence; and current physical activity status. The questions on the gymnastics program related to the following: reasons for adhesion (joining) and adherence (permanence); meaning for the students; and strengths and weaknesses. The questions on general issues related to the following: quality of life; sport and leisure facilities; and autonomy in performing physical activity. The sessions were recorded and later transcribed literally, ensuring the reliability of the data, and the students’ names were replaced by numbers. A battery-operated Sony Recorder, model ICD-PX240, was used, and the audio was transferred directly to the computer via a universal serial bus (USB) cable.

### 2.5. Data Analysis

For the statistical analysis of the WHOQOL-bref questionnaire, the tool provided by Pedroso et al. [12], in their study entitled “Calculation of scores and descriptive statistics of the WHOQOL-bref using Microsoft Excel(Ponta Grossa, Paraná, Brazil)”, was used.

Even though the WHOQOL instruments have been widely used and disseminated, the use of SPSS software to calculate the results is a limiting factor, since it is a paid software. In this sense, aiming to eliminate this limitation, the authors built a tool using Microsoft Excel software to perform the calculation of the scores and descriptive statistics of the WHOQOL-bref questionnaire. It proved to be a reliable tool, which was tested in different versions of Microsoft Excel, and the results returned were compared with those returned by SPSS. The results obtained were identical to those of the syntax proposed by the WHO for SPSS software, in all tested versions of Microsoft Excel [12].

Regarding the analysis of the results of the focus groups, a content analysis technique was used, following Bardin [13], which presents the following criteria for organizing an analysis: pre-analysis, exploration of the material and treatment of the results. In this way, a first reading of the transcripts was performed. In sequence, the themes that appeared in the transcripts of the dialogues were initially categorized and ordered, followed by a specific analysis of each theme, re-reading and scoring the subthemes. The audios transcribed by the researcher are available in Appendix C of the dissertation [6].

### 2.6. Ethical Issues

All participants were informed about the research procedures and gave their informed consent for inclusion before participating in this study. This study was conducted in accordance with the Declaration of Helsinki and the Regulatory Guidelines and Norms for Research Involving Human Beings (Resolution No. 196, of 10 October 1996) of the National Health Council, and the protocol was approved by the Ethics Committee for Research in Human Beings at the USP (School of Arts, Sciences and Humanities) and registered as protocol CAAE: 64651517.6.0000.5390.

### 2.7. Gym Classes

Gymnastics groups began in the mid-1980s and have existed uninterruptedly for about thirty years. To enroll, the student must be a female resident of the city of Valinhos aged between eighteen and fifty-nine years old and present a medical certificate authorizing them to practice physical activity. For people under eighteen, various sports are offered, and for those over sixty, there is a specific program for seniors, but students who turn sixty while attending the classes can continue in the class.

Classes take place twice a week, lasting one hour each, at specific public spots in the city, with the intention being that the student takes the class in their own neighborhood or in a neighboring one but always close to home, preventing the need for a means of transport. This means that only the teacher travels to the location, allowing greater adherence and lower absenteeism. The classes include localized, functional and aerobic exercises, circuit training, and stretching and flexibility exercises. There are also thematic and commemorative classes, walks, open classes with all groups, a June party with square dancing, an end-of-year get-together with a secret friend game and excursions.

## 3. Results

Initially, in this section, the results of the analysis of the WHOQOL-bref questionnaire will be presented, with the aim of characterizing the quality of life of gymnastics students and what quality of life means for them. Next, the results of the analysis of the two focus groups will be presented, and each subtopic corresponds to one of the main subjects approached in its realization: physical activity practices in childhood and adolescence; current physical activity practices; reasons for joining the gymnastics program; reasons for adherence to the gymnastics program; meaning of participation in the gymnastics group; strengths and weaknesses of the gymnastics program; public sports and leisure facilities used for physical activity; physical activities performed autonomously.

### 3.1. Characterization of the Gymnastics Students’ Quality of Life

Table 2 presents the descriptive statistics of the WHOQOL-bref questionnaire for all of the gymnastics students, referring to the tabulation results of all valid questionnaires. Below are, separately, the results of the gymnastics students from Neighborhood A and Neighborhood B, with Neighborhood B being socioeconomically more favored than Neighborhood A. In the tables, the scales are presented in scores that vary from 0 to 20, and on graphs from 0 to 100.

The analysis of Table 2 demonstrates that the greatest amplitude in the responses occurred in the social relations domain, with the smallest amplitude in the psychological domain. The highest averages occurred in the physical (16.00) and QoL self-assessment domains (15.91), while the lowest occurred in the environment domain (13.87). The general average for gymnastics students was 15.23. It can also be observed that, in descending order of the scores, the sequence was: physical, QoL self-assessment, social relations, psychological and environment. 

Table 3 presents the descriptive statistics of the WHOQOL-bref questionnaire for gymnastics students from Neighborhood A. It can be seen that the greatest amplitude in the responses occurred in the environment domain, with the smallest amplitude in the physical domain. The highest averages occurred in the QoL self-assessment (16.14) and physical domains (15.59), while the lowest average occurred in the environment domain (13.87). It can also be observed that, in descending order of the scores, the sequence was: QoL self-assessment, physical, social relations, psychological and environment, similar to the general scores of the students. However, the overall average for these students was 14.84, slightly below the overall average for the students presented above.

Table 4 presents the descriptive statistics of the WHOQOL-bref questionnaire for gymnastics students from Neighborhood B. It can be observed that the greatest amplitude in the answers occurred in the social relations domain, with the smallest amplitude in the psychological domain, similar to the general results of the students. The highest averages occurred in the physical (16.55) and QoL self-assessment domains (16.52), while the lowest occurred in the environment domain (13.93), similar to the general results of the students with only the values of the scores changing. It can also be observed that, in descending order of the scores, the sequence was: physical, QoL self-assessment, psychological, social relations and environment. However, the overall average for these students was 15.51, slightly above the overall score presented above.

According to the analyses presented, in the three cases, the domain with the highest score was the physical domain, while the domain with the lowest score was the environment domain.

Table 5 presents a comparison of the averages of the WHOQOL-bref descriptive statistics among all the subjects, Neighborhood A subjects and Neighborhood B subjects. It can be observed that the average of all research subjects was 15.23, while that of Neighborhood A (14.84) was slightly lower and that of Neighborhood B (15.51) was slightly higher, which also had the highest average for QoL self-assessment. This indicates that the socioeconomically favored group (Neighborhood B) obtained an average quality of life above the overall average of the groups, while Neighborhood A obtained a lower average than the overall average. This demonstrates that, although the difference in means is small between the groups, socioeconomic differences are also reflected in the individuals’ quality of life, which relates to the social determinants of health. 

These are the non-medical factors that influence health outcomes, the social conditions in which people are born, grow, work, live and age, and the broader set of forces and systems that shape the conditions of daily life. These forces and systems include policies and economic systems, development agendas, social norms, social policies and political systems. They have an important influence on health inequities, which are the unfair and avoidable differences in health status observed within and between countries. In this sense, health and disease follow a social gradient: the lower the socioeconomic position, the worse the health [14].

Regarding the meaning of quality of life for the gymnastics students, in Neighborhood A, the main answers were good relationship with family members and people around them and the practice of physical activity, but there were also four mentions for the practice of physical exercise, as well as having a good relationship with the people around them, including the importance of a good family coexistence, to the quality of life. There were also two citations for leisure being important for quality of life, having good physical health and healthy aging, as well as citations linking the concept with good nutrition; however, they did not mention the term healthy eating. This term relates to the condition of being financially capable of having a good diet, in the sense of having purchasing power to buy healthy and balanced food. There was also only one answer for the relationship between quality of life and financial situation, such as having a job and income that makes it possible to buy a home, the right to housing, healthy habits, access to education and independence in the activities of daily living.

As for Neighborhood B, it can be seen that the main responses were healthy eating (five students), physical activity (four students) and being in good health (two students). Regarding healthy eating, one of the students also mentioned the possibility of eating fruits and vegetables, in the financial sense, and there was only one citation for the following: feeling good about oneself, praying, meditating, the population’s access to fundamental rights, such as health and education, having independence in activities of daily living, leisure, everything that is good for the individual and having the will to do everything (psychological health).

The interaction between living conditions and health has been widely indicated throughout history [15], and the study of individuals’ quality of life has become a prominent topic in contemporary society; however, research involving this subject should consider that this is a complex topic, because it involves objective and subjective aspects, conditions and lifestyles, as well as multidimensional factors [16].

As observed in the responses, it is a broad concept, and as it also has an individual parameter, it can vary from one individual to another, which explains some of the different responses within and between the groups. However, even with different answers, both groups essentially agreed on the importance of physical activity to the quality of life, as well as healthy eating, good health and leisure activities.

### 3.2. Physical Activity Practices in Childhood and Adolescence

In this section, the physical activity practices in childhood and adolescence of the gymnastics students from Neighborhood A and Neighborhood B are presented in the Table 6.

In Neighborhood A, the students showed excitement when telling their life stories, but some expressed a certain sadness when remembering what they had lived through in childhood, especially in relation to the home environment; at various times, they identified or sympathized with each other’s stories.

Several students reported that they almost did not play because their father was very strict, they were beaten often, they started working very early, they got married very young (in some cases to escape their father’s severity) and they had children when they were still teenagers.

The issue of access to study was also a striking point in this neighborhood, highlighted by the impossibility of continuing their education due to the need to work imposed on the students by their families. In the responses of several students, it was mentioned that they only studied until the fourth year of elementary school.

It is also important to point out that, in some cases, the same game was cited by some students as a school game—generally more like a playground game than school physical education, as several students did not take this subject, and some studied for only a few years—and by others as a game played in the street.

Work activities, such as drawing water from the well and carrying firewood and walking to and from school were also cited as physical activity, in addition to participation in festivities (June festival, carnival and dances) mainly due to the practice of dancing on these occasions; however, some students reported that they participated hidden from their fathers.

They also mentioned that it was rare for someone to have a bicycle at that time, so those who had one would share it with their siblings, cousins or friends. Regarding Neighborhood B, the students also showed excitement when telling their memories of childhood and adolescence, but with slightly more excitement and happiness, with some reporting that their childhood and adolescence were good.

Unlike in the previous group, none of the students mentioned that their childhood was sad, that they would rather not talk about that period, that they almost did not play because they started working too early, that they had to drop out of school because of work or that they got married very young. According to their responses, these students also had a greater opportunity to study, and several reported that they participated in physical education at school, that the teachers were demanding, as it was during the military period, and that the physical exercises were so rigorous that some students even felt sick in the classes.

The issue of male chauvinism in the ban on playing soccer was also mentioned, but to a lesser extent, and in this case, it happened in the school physical education program, as it was during the military period. However, Student 17 reported that she played soccer in a team that represented her city, as well as playing in championships outside her municipality about forty-five to fifty years ago, which was not common at the time, demonstrating her belonging to a socioeconomically privileged group.

Student 14 reported that she had been a member of a club since she was eight years old, and that she used the pool quite often, as it was close to her residence. Additionally, she also mentioned that she used to ride a bike frequently.

In Neighborhood B, the students also commented that it was difficult for someone to own a bicycle, as bicycles were very expensive at the time, but they did not comment on sharing a bicycle. Likewise, some students carried out work activities in the fields, but did not report them as physical activity. Furthermore, activities related to festivities were not mentioned, but some toy constructions were mentioned, which did not occur in the previous group, as well as school physical education during primary school.

### 3.3. Current Physical Activity Practices

Regarding current physical activity practices, in addition to gymnastics classes, in Neighborhood A, the most common practices were walking (seven students) and weight training (three students), but there were also two mentions for playing with grandchildren, Zumba, walking around town, water aerobics, dancing at senior dances and housework, and just one mention for swimming, child care, Lian Gong, walking a lot during work, running and walking intervals, and riding a roller cart. An interesting finding is that housework and taking care of children were mentioned as physical activity, due to the physical fatigue they cause.

Regarding Neighborhood B, the most common practices were walking (four students) and ballroom dancing (two students), but there was also a citation for localized gymnastics and stretching following a cassette tape, running, climbing trees, riding a bicycle, bodybuilding, Zumba and dance fitness.

Thus, the main physical activity performed by the students in both groups, with the exception of gymnastics, was walking.

### 3.4. Reasons for Joining the Gymnastics Program

Regarding the reasons for joining the gymnastics program, in Neighborhood A, the main reasons were medical advice (five students) and invitation from members (four students). Two students mentioned that they signed up for both reasons together, and one student did not respond. Student 7 reported that, in addition to her friend’s invitation, she wanted to improve her daily life activities, and Student 11 reported that she likes to perform physical activity. Student 9 mentioned that she returned to the group after a period of absence, because she loves the teacher so much; this affection was a remarkable observation during the focus groups.

Regarding the students from Neighborhood B, the main reason for joining was invitation from members (four students). Only Student 18 started the class due to medical advice. Student 16 tried bodybuilding but did not like it. Student 14 aimed to improve her physical and psychological health. Student 13 was one of the citizens responsible for starting gym classes in the neighborhood in 1996, which demonstrates the social control of public sport and leisure policies in the city of Valinhos.

### 3.5. Reasons for Adherence to the Gymnastics Program

Concerning the reasons for permanence, analyzing the individual responses from Neighborhood A, it was possible to verify that the reasons most cited by the students were the enjoymentof participating in the group (five students), having fun during classes (five students) and the possibility of leaving home (four students). There were also three answers for taking care of one’s health, feeling less physical pain and making friends.

It should be noted that there were no results related to aesthetics or weight loss. Most responses referred mainly to the gym class as a sociability space, that is, a moment of meeting, exchanges or social contributions between friends inthe group. This was the main reason for the students’ permanence in the gymnastics group in Neighborhood A. The answers related to this idea were the enjoyment of participating in the group, having a laugh or having fun during classes, the possibility of leaving home, making friends, being well received by the teacher and the students in the class, feeling less ashamed to speak in public after participating in the group, having a moment of relaxation, enjoying the festivities during classes, leaving the routine at home, taking time to meet friends, the possibility of having friendships, enjoying the friendship of the teacher and the students, the possibility of talking to other people and learning new things.

Some answers related to health, both physical and psychological, were also presented, and one student reported that the place is easily accessible, as it is close to her home. The responses related to physical health were feeling less physical pain, feeling less cramps during sleep, feeling less physical fatigue and indisposition in everyday life, improving conditioning and disposition in daily activities, especially domestic ones, maintaining the amplitude of movements of the joints and postural correction. With regard to psychological health, the responses were feeling good about participating in classes, it is good for the mind and soul, feeling good about participating in the group, stress control and the possibility of working on emotions. The students also showed great affection for the teacher.

With regard to Neighborhood B, the reasons for adherence most cited by the students were liking the teacher (seven students), enjoying participating in the classes (six students), liking the group (five students) and liking the friendships (four students). There were also three answers for providing well-being and enjoying trips and tours, and two answers for feeling less physical pain. In this group, the students also showed great affection for the teacher.

It is important to point out that in this group, there were also no results related to aesthetics or weight loss, and, as in Neighborhood A, most of the responses referred mainly to the gym class as a sociability space. This was also the main reason for the adherence of the students to the gymnastics group in Neighborhood B, and the answers related to this idea were that it is already part of life, liking the group, the friendships, the trips, the tours, the festivities during the classes and the moments of exchange. Some responses related to health were also presented, such as the possibility of taking care of the body, health and well-being. With regard to physical health, it was mentioned that the students felt less pain in their body, and in terms of psychological health, an improvement in depression and well-being was provided by the classes.

To finalize this question, it is important to report that in both focus groups, the main reason for the adherence of the students was the understanding of the gym class as a sociability space, transforming the participation in the classes into a moment of meeting and exchanges (interpersonal relationships), as well as the possibility of leaving home, making new friends, talking to different people, having fun and belonging to a group. For some, this represents the only possibility since most of the students are housewives. In the background, they also aim to take care of their physical health—mainly to feel less body pain—and psychological health. In addition, it can be seen that participation in gym classes represents a moment of leisure, and this is also an important health promotion strategy.

### 3.6. Meaning of Participation in the Gymnastics Group

Regarding what it means for students to participate in the gymnastics group, analyzing the individual responses from Neighborhood A, it was possible to verify that the main responses were the group means family (four students), and the group means everything in the life of the student (three answers). There were also two answers for the following: possibility of leaving home, making friends, meeting friends, having fun and taking care of one’s health. Additionally, there was just one answer for feeling better physically and psychologically, a moment of relaxation, the possibility of participating in a group, having friends, feeling less shy about interacting with other people, happiness at being able to talk to other people, a moment of laughing and learning new things.

Regarding Neighborhood B, the main answer (four students) was that participation in the gymnastics group means a moment of exchanges and sociability among the students, which helps in promoting health, especially psychological health. For example, Student 20 reported that their depressive condition was improved by participating in the gymnastics group. There was also only one answer for the possibility of meeting different people and liking them, a moment of meeting the other students, relieving stress and occupying one’s mind. The group means a lot because of the friends and everything that happens in the classes, such as trips, and because it improves their health and provides a family, becoming a part of the student’s life (who can no longer live without participating in the gymnastics group). For the students, there is nothing better; the group is very good because the students and the teacher are nice, and it provides the possibility to explore one’s “inner self”, and improves depression, emotions and life itself.

To finish this point, it can be said that for the gymnastics students from Neighborhood A, the group represents a family, and for the students from Neighborhood B, the group offers a moment of sociability. In both groups, the classes positively influence quality of life, mainly through the psychological and social benefits provided by the practice of group-oriented physical activity, which is directly related to the reasons for their permanence in the gymnastics group.

In view of this, in addition to physical health, it is possible to state that the motivations for the practice of physical activities involve the daily interactions made possible by new collegial relationships and even friendships established throughout the activities, the search for well-being and for new knowledge, and the perspective of improving the quality of life through established bonds and shared experiences [17].

### 3.7. Strengths and Weaknesses of the Gymnastics Program

With regard to the strengths of the gymnastics modality, as a municipal public policy of the SEL, the main positive point reported by the students from Neighborhood A was that the gymnastics class was addressed to them, which they appreciated, considering it good, active and appealing, along with the well-being provided by its practice. There were also two answers for the friendships among the students, and one answer for seeing results, an easily accessible place because it is close to home, the teacher’s dedication and studies, the possibility of healthy aging and changing one’s mind when participating in the classes. With regard to the gymnastics group in Neighborhood B, the main positive point reported by the students was the teacher, where they praised her professionalism, her dedication, the level of demand and the intensity of the class. Two students also mentioned the friendships among the group.

As for weaknesses, the main issue addressed by the students from Neighborhood A was the desire for more class days per week. They also mentioned the lack of a substitute duringthe teacher’s vacation period. Meanwhile, three students stated that there were no weaknesses. In Neighborhood B, the main answer was that there were no weak points. However, three students mentioned that they would like more days of class per week, and another three students reported that the weak point was when they themselves missed class.

### 3.8. Public Sport and Leisure Facilities Used for Physical Activity

With regard to public sport and leisure facilities, the students from Neighborhood A agreed that there is good will on the part of the municipal administration and that there are many good classes taking place but that the city needs more public facilities for autonomous practice, including spaces for walking and bicycle lanes. In addition, it was reported that there are public spaces that are underutilized for classes, such as sports courts that need repairs.

In Neighborhood B, it was verified that the public facility most used by the students is the Worker’s Leisure Center (Centro de Lazer do Trabalhador, CLT), a park that has an indoor and outdoor walking track, with mileage tracking, facilitating the practice of walking, as well as a runway. It also has an indoor bicycle lane, an outdoor gym and a functional outdoor gym for the elderly, and it is close to their homes. Of the eight participants in the focus group, five declared that they use it frequently. One student mentioned Taquaral Lagoon Park (Parque Lagoa do Taquaral) in Campinas (a neighboring city), and another reported that she sometimes goes to the 500 Years Square (Praça 500 anos) where it is possible to walk and use the outdoor gym. They also mentioned Washington Luiz Square (Praça Washington Luiz) and two other outdoor gyms (on Andradas Highway and Paulista Avenue) although they rarely attend them, preferring the CLT due to its structure and greater proximity.

As for bicycle lanes, the students commented that they would be interesting for the city, but it is necessary to first make drivers aware of respect for cyclists, and it may also not be advisable throughout the municipality as the city has many hills which would make commuting by bicycle difficult in these locations. In addition, they reported the existence of only one public swimming pool in the city, which entails a long waiting list and great difficulty in obtaining a vacancy; however, they mentioned the existence of a project to build a public swimming pool in their neighborhood.

Finally, the facility most used by Neighborhood B is the CLT, which guarantees not only the practice of physical activity but also a moment of leisure in a green area within the city. Urban parks, as important leisure spaces, have their current configuration due to the intense changes that cities experienced from the 19th century onwards, with the processes of industrialization and urbanization. They contain elements of the countryside, appearing as refuges in the city and enabling the urban society to escape from the hardships of the industrial city [5].

### 3.9. Physical Activities Performed Autonomously

Regarding the practice of physical activities performed autonomously by the students, that is, not guided, in Neighborhood A, the most mentioned activity was stretching (six students), followed by postural correction (four students) and sit-ups (three students). There was only one citation for teaching gym class exercises to an adult son and doing home gymnastics with a granddaughter.

As for Neighborhood B, the main citation was postural correction (six students), followed by stretching (five students), using the CLT outdoor gym (two students) and running (one student).

In this way, in both neighborhoods, the main physical activities carried out autonomously by the students were stretching and posture correction in everyday life, both in the practice of physical activities and in carrying out housework and work activities, with the aim of feeling less pain in the spine.

## 4. Discussion

This study’s main objective was to understand the reasons for students’ adhesion and adherence to gymnastics groups, in order to understand why this public policy has been taking place uninterruptedly for over thirty years and to characterize the students’ quality of life. The secondary objectives were to identify what this participation means for them, to understand the strengths and weaknesses of the gymnastics program, to identify what quality of life means for the students, and whether they use other sport and leisure facilities or carry out other guided or autonomous physical activities.

When assessing the quality of life of the SEL gymnastics students, it can be seen that the socioeconomically favored group (Neighborhood B) obtained an average quality of life above the general average of all the groups, while Neighborhood A obtained a lower average, demonstrating that social and economic differences can also be reflected in quality of life. It was also possible to verify the difference in the meaning of quality of life between the two classes and between students of the same class. This agrees with the WHO, which defines quality of life as an individual’s perception of their place in life, in the context of the culture and value systems in which they live and in relation to their goals, expectations, standards and concerns. It is an extensive and complex concept, which encompasses physical and psychological health, the level of independence, social relationships, personal beliefs and relationships with the characteristics of the environment. This definition reveals criteria which state that quality of life refers to a subjective assessment, with positive and negative dimensions, and that it is rooted in a cultural, social and environmental context [18].

Likewise, it reflects the perception among individuals that their needs are being met, or that opportunities to achieve happiness and self-fulfillment are being denied, regardless of their physical health status or social and economic conditions [18]. Additionally, as such, it can be said that the concept of quality of life is subjective, complex and multidimensional [9], as observed in the students’ responses in the focus groups.

During the realization of the focus groups, it was also possible to observe these socioeconomic differences between the two studied groups, in the students’ answers and mainly in their memories of childhood and adolescence, both in the reports of what family life was like, and in access to study and physical activity and leisure practices.

However, despite these differences reflecting opportunities to experience physical activity from childhood to the present moment, including access to public sport and leisure facilities close to the residence, the reasons for joining and adhering to the gymnastics group were similar in both neighborhoods. Additionally, when carrying out the two focus groups, the need for the participants to talk about life episodes that were not foreseen in the script was observed; therefore, through the present study, it was possible to give voice to these women who felt welcomed by the researchers.

Regarding the reasons for joining, it can be said that in both groups, the students joined primarily due to the indication of practicing students, whether they were relatives, friends or neighbors, and due to medical indications. 

Afterwards, it was possible to understand that, when starting to attend gym classes, their initial objectives were contemplated and they were faced with a new reality, that of belonging to a group and of perceiving the gym class as a space of sociability. For the students, the group became a moment of encounters, interpersonal relationships and social support, which led to low turnover in the classes and the students’ permanence in the group for many years, regardless of their social class.

These findings coincide with other studies, showing that, currently, the reasons for the practice of physical activity, such as sport, isthat it is part of the social life of individuals and, among the countless biological benefits provided, is increasingly sought after for the sensation of pleasure and well-being it provides. Health, as a concept, is also based on human and social sciences [19]. However, the complexity of health is undeniable, regardless of the perspective from which it is approached [20]. A study carried out by Meurer, Benedetti and Mazo (2012) showed that health, pleasure and sociability are the main reasons for staying in a physical activity program [21].

In the same sense, Conceição et al. (2011) [22] analyzed the reasons for joining and permanence in a physical activity program for 112 elderly people. The most cited reasons for joining the physical activity program were medical indications; to maintain/improve health; and the need to do physical activity (for pleasure and to feel good). With regard to the reasons that led to the elderly participants’ permanence in the physical activity program, the following stood out: feeling good by regularly practicing physical exercise; their health improvement/maintenance; and the relationships/social coexistence that the program makes possible. However, in one point, their study differs from the present study, because the general indication from friends, neighbors and/or family was one of the least cited reasons for admission, contrary to our finding. However, in a study by Cardoso et al. (2008), it was concluded that the main reason for joining the physical exercise program was the relationship aspect, that is, invitations from friends and/or family members [23].

Therefore, a new study would be necessary to understand specifically why the indications of students already participating in the program have such an influence on the adhesion of new students. We believe that this may be related to what it means for the students to participate in their gymnastics group. With regard to the meaning of participating in the gymnastics group, the results demonstrated that for the students in the Neighborhood A gymnastics group, the group represents a family for them, and for the gymnastics students of Neighborhood B, the group represents a moment of sociability. In both cases, it is worth concluding that the group has a positive influence on the quality of life, through the psychological and social benefits provided by the practice of group-oriented physical activity. In the same way, it was verified that these meanings are directly related to the reasons for their adherence in the gymnastics groups.

As presented by Kim, Lee and Kim (2021), several existing studies have examined the effects of exercise. Among them, it can be said that group exercise programs improve social interaction, and exercise improves cognitive functions and depressive symptoms [24]. According to Silva et al. (2010), among the differences in the quality of life of people who practice physical activities compared to those who do not, there are not only physical health aspects but also psychological and cognitive aspects [25]. According to Conceição et al. (2011), coexistence and social relationships stood out as relevant factors for continuity in the physical activity program [22]—in other words, sociability. For Mathias et al. (2019), in the practice of physical activity, the sociability dimension concerns the opportunity to live with friends and obtain new friends [26].Another point observed during the students’ responses was that gym classes also mean a moment of leisure. In this sense, leisure is presented as a human need which the students satisfy through their participation in classes. Combined with the fact that this moment entails benefits in the quality of life, it can be said that leisure is also configured as a health promotion strategy.

Physical activity, as a phenomenon oriented towards the subject’s achievement, seems to be a domain in which society has gained greater awareness, being associated with what is favorable and consistent with the highest aspects of the individual’s quality of life, that is, their well-being. In contemporary society, there is also a very marked connection between physical activity and leisure, or a culture of the body in leisure activities, which cannot be dissociated from the concept of health or from the set of references of quality of life and well-being associated with it [27].

As presented by Batista, Ribeiro and Nunes Junior (2012), when a practice is chosen, experiences that are pleasant and more suitable for the use of free time are chosen. In addition, the choice of a certain experience not only achieves biological benefits, such as physical conditioning and regulation and strengthening of metabolic systems, but also contributes to other social determinants, such as sociability, balanced use of time and access to culture, which will certainly trigger results in maintaining health as a whole [28].

In addition, with the aim of expanding the offer of physical and leisure activities in the city, it is important to mention the possibility of associating oriented programs with the stimulation of autonomous physical activity, through programs that encourage these practices, as well as the improvement of existing sport and leisure facilities and the availability of new facilities, in different locations in the municipality, in order to expand the reach of the population, which will allow the expansion of health promotion actions in the city and greater democratization of spaces and practices. Together, there must be a constant evaluation of municipal public policies, so that they are rethought, adapted or replaced, when necessary, with a focus on the best possible service for citizens in projects and programs of the various secretariats, considering the quality of life as a broad and multifaceted concept, which also demands intersectional actions and interdisciplinary projects.

Another important point that cannot be forgotten is that the practice of physical activity also improves mental health, which relates to a significant current need of humanity, mainly due to the increase in or worsening of cases of mental disorders caused by the COVID-19 pandemic [29,30,31]. In particular, in developing countries such as Brazil, public health systems are overwhelmed, as reported by Bastos et al. [32], and patients are often on a long waiting list for health care. Therefore, it is important that other integrative and complementary practices, such as physical activity, be associated both in the prevention and treatment of comorbidities, as an alternative to relieve the public system and guarantee greater access to health for the population since public policies are essential for social rights to be contemplated, hence the importance of studying and discussing public policies on physical activity and their relationship with the expansion of health promotion actions through active behavior.

The limitations of this study are as follows: the sample consisted only of women; a control group was not used to compare the results of the WHOQOL-bref questionnaire, since the main focus of this study was to analyze the reasons for joining and adherence to the gymnastics program; a comparison of these motives between the most recent students and those who had been participating the longest was also not carried out; this study also presented many objectives to be achieved, since the intention was to understand all the reasons that have led to the gymnastics program existing for so long, even though it is a municipal public policy, which is not very common in Brazil. However, despite these limitations, we believe that this article presents relevant data to serve as an example for the creation of future public policies, mainly by demonstrating that the practice of physical activity not only promotes benefits in physical health but also represents an important strategy for the expansion of health promotion actions.

## 5. Conclusions

This study proved that the practice of gymnastics as a municipal public policy led to an improvement in the physical and mental health of the students, as well as positively influencing several facets of their quality of life, which further emphasizes the importance of public policies as catalysts for health behavior and guarantees of constitutional social rights, such as access to health, sport and leisure. These policies need to be inclusive, democratic and designed according to the needs of those served; therefore, it is essential to understand the reasons for adhesion and adherence to a program, in order to improve the program or use it as an example for the creation and elaboration of future policies or programs.

In addition to reducing sedentary behavior and improving biological indicators, which are extremely important for health, mainly because they prevent chronic non-communicable diseases, which overload health systems, physical activity also provides social and psychological benefits, which positively influence the quality of life of the practitioners. This also enables physical activity to be characterized as a health care strategy, from a biopsychosocial perspective, which is a topic that needs to be addressed in undergraduate courses, continuing education courses and postgraduate courses in the field of physical education. 

## Figures and Tables

**Table 1:** Background information of the focus group participants.

Group	Student	Age (years)	Adherence
Neighborhood A	1	60	2 years
	2	44	5 months
	3	46	5 months
	4	47	7.5 years
	5	63	12 years
	6	69	30 years
	7	60	30 years
	8	49	2 years
	9	57	5 years
	10	68	3 years
	11	52	2 years
	12	55	4 years
Neighborhood B	13	65	23 years
	14	54	3 years
	15	55	6 years
	16	46	2 years
	17	63	21 years
	18	63	21 years
	19	61	5 years
	20	65	24 years

**Table 2:** WHOQOL-bref descriptive statistics for SEL gymnastics students.

Domain	Average	Standard Deviation	Variation Coefficient	Min Value	Max Value	Amplitude
Physical	16.00	2.05	12.80	8.57	20.00	11.43
Psychological	15.63	1.92	12.30	10.00	20.00	10.00
Social relations	15.79	2.61	16.51	5.33	20.00	14.67
Environment	13.87	2.00	14.43	7.50	20.00	12.50
QoL self-assessment	15.91	2.35	14.76	8.00	20.00	12.00
Total	15.23	1.66	10.87	9.69	19.69	10.00

**Table 3:** WHOQOL-bref descriptive statistics for gymnastics students from Neighborhood A.

Domain	Average	Standard Deviation	Variation Coefficient	Min Value	Max Value	Amplitude
Physical	15.59	1.88	12.06	13.71	20.00	6.29
Psychological	15.10	2.32	15.36	10.67	19.33	8.67
Social relations	15.43	2.60	16.85	8.00	17.33	9.33
Environment	13.43	2.91	21.70	10.00	20.00	10.00
QoL self-assessment	16.14	2.54	15.72	12.00	20.00	8.00
Total	14.84	2.18	14.67	11.36	19.54	8.18

**Table 4:** WHOQOL-bref descriptive statistics for gymnastics students from Neighborhood B.

Domain	Average	Standard Deviation	Variation Coefficient	Min Value	Max Value	Amplitude
Physical	16.55	2.09	12.60	10.29	19.43	9.14
Psychological	16.14	1.39	8.62	14.67	20.00	5.33
Social relations	15.30	3.11	20.33	5.33	20.00	14.67
Environment	13.93	1.77	12.72	10.50	18.00	7.50
QoL self-assessment	16.52	1.83	11.08	12.00	20.00	8.00
Total	15.51	1.59	10.26	12.00	18.15	6.15

**Table 5:** Comparison of averages of WHOQOL-bref descriptive statistics for SEL gymnastics students between Neighborhoods A and B.

Domains + QoL Self-Assessment Averages	All Groups Average	Neighborhood A Average	Neighborhood B Average
Physical	16.00	15.59	16.55
Psychological	15.63	15.10	16.14
Social relations	15.79	15.43	15.30
Environment	13.87	13.43	13.93
QoL self-assessment	15.91	16.14	16.52
Total	15.23	14.84	15.51

**Table 6:** Physical activity practices in childhood and adolescence of gymnastics students from Neighborhood A and Neighborhood B.

Physical Activity Practices	Neighborhood A	Neighborhood B
**Games played at school**	Yard/playground: hopscotch, ball play, rope jumping, dodgeball and ciranda.	Physical education: running, sit-ups, stretching, gymnastics, handball and volleyball (soccer was only for boys); only Student 19 played soccer at school.
**Games performed on the street**	Hopscotch, tag, hide and seek, playing ball (sometimes hidden from the father), riding a bicycle, playing discus, dodgeball, bat/bets, jumping rope, playing shuttlecock, ciranda and bocce.	Dodgeball, soccer, dog tag, hide and seek, hopscotch, jumping rope, doing somersaults, walking on stilts and playing shuttlecock.
**Invented games**	Hurricane (rolling down the street inside a tire), jumping across the river with a pole, floating in floodwaters inside a basin with another person pushing, bean straw hut and bonfire.	Jumping in the river with a bamboo pole and going down the hills sitting on cardboard.
**Physical activities related to nature**	Swimming in a river, horseback riding, climbing trees and fishing with a sieve.	Climbing fruit trees.
**Other physical activity practices**	Physical activities related to festivities: participating in ball (some hidden from the father), June parties, carnivals, samba and matinees.	Invented toys: playing with clay, making little animals by putting sticks in chayote and making dolls out of green corn on the cob.
	Labor activities: drawing water from the well and carrying firewood.	Walking to and from work.
	Walking to and from school.	Walking to and from school.
	Playinghouse.	Riding a bicycle.
	Swing (playground swing).	Swimming in the club’s pool.
	Rodeo (rotating toy found in a children’s playground).	
	Dancing.	

## Data Availability

Not applicable.
